# Supplementary material for: Dynamic SARS-CoV-2 emergence algorithm for rationally-designed logical next-generation vaccines
Source: Commun Biol. 2022 Oct 10;5:1081. doi: 10.1038/s42003-022-04030-3 (PMC9550860; doi:10.1038/s42003-022-04030-3)
Supplement: Supplementary file 2 — Supplementary Information [file 42003_2022_4030_MOESM2_ESM.pdf]

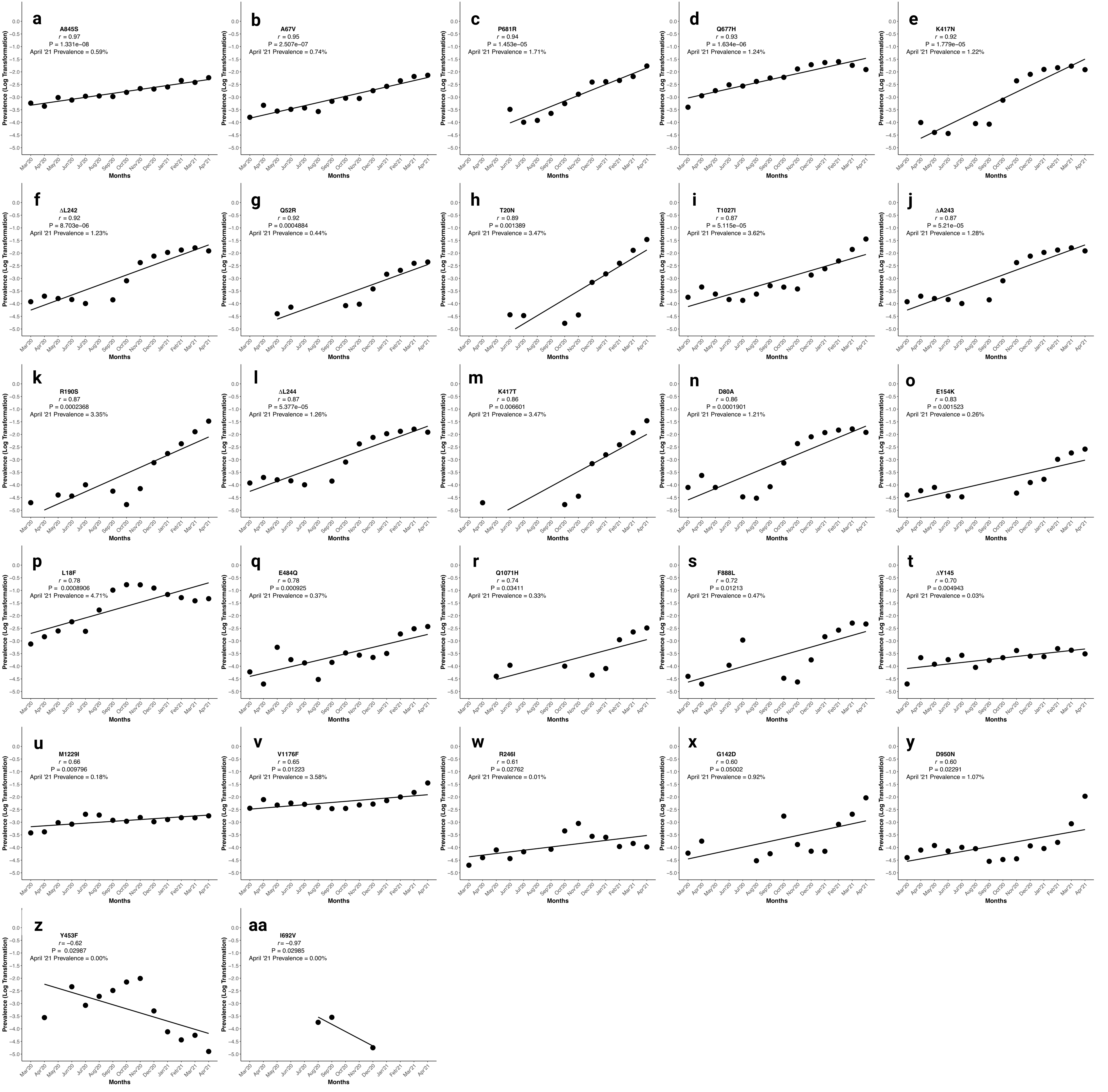

Supplementary Figure 1. Pearson's Correlation on Logarithmically-Transformed Prevalence Ratios of the Remaining SARS-CoV-2 Variant Amino Acid Substitutions and Deletions Not Currently Selected via the Algorithm. This figure shows the graphical representation of the SARS-CoV-2 spike protein amino acid substitutions and deletions not currently concerning due to low previous month prevalence, low *r* value, or insignificant P value. Though not yet of concern as of April '21, those substitution and deletions represented by high *r* values should be cause for close monitoring. Each graph denoted by an alphabetical character or characters represents a unique amino acid substitution or deletion in the spike protein of SARS-CoV-2 (a) A845S, b) A67V, c) P681R, d) Q677H, e) K417N, f) ΔL242, g) Q52R, h) T20N, i) T1027I, j) ΔA243, k) R190S, l) ΔL244, m) K417T, n) D80A, o) E154K, p) L18F, q) E484Q, r) Q1071H, s) F888L, t) ΔY145, u) M1229I, v) V1176F, w) R246I, x) G142D, y) D950N, z) Y453F, and aa) I692V). Graphs were generated using open-source RStudio version 1.3.1093 (R version 4.0.3) and the ggplot2 package under MIT + license (https://cran.r-project.org/web/packages/ggplot2/index.html). Graphs were compiled and the final figure generated using Biorender.com.

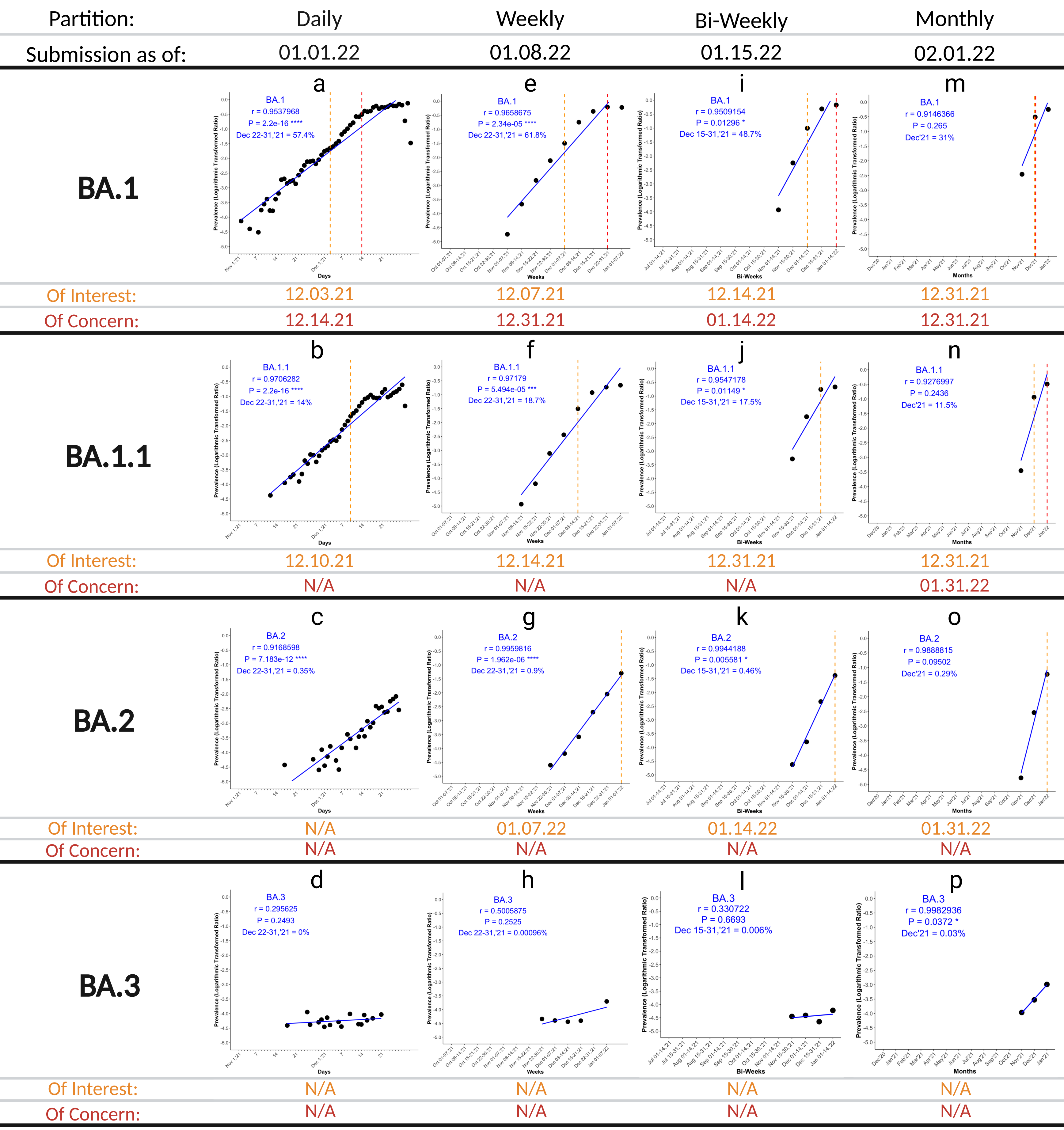

Supplementary Figure 2. Pearson's Correlation on Logarithmically-Transformed Prevalence Ratios of the Omicron SARS-CoV-2 Variant of Concern Sublineages. This figure demonstrates the quantitation of the sublineages of the Omicron SARS-CoV-2 Variant of Concern. The emergence is shown for each of the sub-lineages of the Omicron VOC (BA.1 (a, e, i, m), BA.1.1 (b, f, j, n), BA.2 (c, g, k, o), and BA.3 (d, h, l, p)). The partitions are demonstrated in daily partitions (a, b, c, d), weekly partitions (e, f, g, h), bi-weekly partitions (i, j, k, l), and the prototype monthly partition (m, n, o, p). Each lineage and partition displays the date the VOC would be classified "of interest" (orange) and "of concern" (red) as defined by the Algorithm. Additionally, the cut-off date for submissions demonstrates when the classification would occur. *p <0.05, **p<0.005, ***p<0.0005, ****p<0.00005. Graphs were generated using open-source RStudio version 1.3.1093 (R version 4.0.3) and the ggplot2 package under MIT+ license (https://cran.r-project.org/web/packages/ggplot2/index.html). Graphs were compiled and the final figure generated using Biorender.com.

Supplementary Table 1. Genetic Characteristics of the Hawaii SARS-CoV-2 Variant B.1.243 Isolates, USA-HI498 2020* and USA-HI708 2020**

Gene	Nucleotide			Amino Acid		
	**Loci**	**Wild Type**	**Mutant**	**Loci**	**Wild Type**	**Mutant**
5' UTR	241	C	T	-	-	-
ORF1ab	3,037	C	T	924	Phe (F)	Phe (F)
ORF1ab	10,741	C	T	3492	Asp (D)	Asp (D)
ORF1ab	12,076***	C	T	3937	Asn (N)	Asn (N)
ORF1ab	14,408	C	T	4715	Pro (P)	Leu (L)
ORF1ab	20,268	A	G	6668	Leu (L)	Leu (L)
S	23,403	A	G	614	Asp (D)	Gly (G)
S	23,604	C	A	681	Pro (P)	His (H)
S	24,076	T	C	838	Gly (G)	Gly (G)
N	28,854	C	T	194	Ser (S)	Leu (L)
N	29,266***	G	A	331	Leu (L)	Leu (L)
3' UTR	29,710	T	C	-	-	-

GenBank accession *MZ664037 and **MZ664038, ***exclusive to SARS-CoV-2, Isolate USA-HI498 2020

Supplementary Table 2. Pearson's Correlation of Logarithmic Transformed Prevalence Ratios of Amino Acid Substitutions (AAS) in the Spike Protein and Variants from March 2020 - April 2021 and Pairwise Heatmap of Absolute Value Difference of *r*-value between Corresponding AAS and Variants

				Variant	P.1 Gamma	B.1.617.2 Delta	B.1.617.1 Kappa	B.1.351 Beta	B.1.1.7 Alpha	B.1.429 Epsilon	B.1.427 Epsilon	B.1.525 Eta	P.2 Zeta	B.1.243 -	B.1.1.298 -	B.1.1 -
				Origin	Brazil	India	India	South Africa	United Kingdom	California	California	United Kingdom	Brazil	Hawai'i	Denmark	Nigeria
				n	12,485	2,758	2,037	14,798	580,502	26,779	12,305	3,411	2,350	9,726	1,533	41,833
				April '21 Prevalence (%)	3.04	1.08	0.35	1.15	68.55	1.49	0.51	0.42	0.04	0.04	0.00	0.35
				P Value	0.00	0.01	0.02	0.00	0.00	0.00	0.00	0.04	0.00	0.50	0.65	0.00
Substitution	n	April '21 Prevalence (%)	P value	r	0.97	0.96	0.94	0.94	0.92	0.92	0.92	0.89	0.83	0.20	0.17	-0.98
E484K	60,990	9.39	0.000	0.98	0.010			0.046				0.093	0.157		0.810	
P681H	641,501	79.02	0.000	0.98					0.053					0.779		1.959
A845S	4,830	0.59	0.000	0.97												1.950
ΔV70	614,413	74.62	0.000	0.96					0.041			0.073			0.790	
ΔH69	613,401	74.49	0.000	0.96					0.040			0.073			0.790	
N501Y	636,491	81.03	0.000	0.96	0.015			0.021	0.036				0.133		0.785	
S982A	594,828	75.05	0.000	0.95					0.025							
A67V	5,784	0.74	0.000	0.95								0.057				
T478K	17,655	2.46	0.000	0.95		0.017										
P26S	18,361	3.66	0.000	0.95	0.029								0.119			
P681R	8,571	1.71	0.000	0.94		0.022	0.002									
L452R	56,039	5.44	0.000	0.94		0.026	0.002			0.020	0.020					
A701V	35,639	5.12	0.000	0.93				0.004								
D1118H	595,048	75.21	0.000	0.93					0.009							
W152C	37,487	2.12	0.000	0.93						0.012	0.012					
Q677H	23,205	1.24	0.000	0.93								0.037				
T716I	608,252	75.99	0.000	0.93					0.004							
A570D	609,134	76.71	0.000	0.93					0.002							
T95I	31,010	6.06	0.000	0.92			0.016									
K417N	15,367	1.22	0.000	0.92				0.015								
H655Y	18,155	3.89	0.000	0.92	0.055								0.093		0.746	
ΔL242	14,464	1.23	0.000	0.92				0.019								
Q52R	3,138	0.44	0.000	0.92								0.026				
S13I	33,379	2.02	0.000	0.92						0.001	0.001					
D138Y	19,518	3.64	0.000	0.92	0.058								0.089		0.742	
ΔY144	600,274	75.49	0.000	0.90					0.026			0.006				
T20N	13,656	3.47	0.001	0.89	0.087								0.061			
T1027I	14,941	3.62	0.000	0.87	0.104								0.043			
ΔA243	15,132	1.28	0.000	0.87				0.068								
R190S	13,441	3.35	0.000	0.87	0.105								0.042		0.695	
ΔL244	14,735	1.26	0.000	0.87				0.069								
K417T	13,226	3.47	0.007	0.86	0.118								0.029		0.682	
D80A	15,126	1.21	0.000	0.86				0.083								
E154K	1,505	0.26	0.002	0.83			0.109									
L18F	91,123	4.71	0.001	0.78	0.190								0.043			
E484Q	2,447	0.37	0.001	0.78			0.157									
Q1071H	1,783	0.33	0.034	0.74			0.196									
F888L	3,698	0.47	0.012	0.72								0.170				
ΔY145	482	0.03	0.005	0.70					0.220			0.188				
D614G	1,446,840	99.24	0.009	0.67	0.306	0.295	0.271	0.270	0.255	0.249	0.249	0.223	0.158	0.470	0.494	1.650
M1229I	2,058	0.18	0.010	0.66											0.488	
V1176F	19,419	3.58	0.012	0.65	0.327								0.179			
R246I	290	0.01	0.028	0.61				0.331								
G142D	3,190	0.92	0.050	0.60			0.338									
D950N	2,895	1.07	0.023	0.60		0.363										
K150N	180	0.04	0.040	0.60												
V308L	1,212	0.08	0.060	0.51												
Y453F	1,689	0.00	0.030	-0.62											0.799	
I692V	19	0.00	0.030	-0.97											1.145	

Supplementary Table 3. Comparison of Single Nucleotide Polymorphisms (SNP) and Resultant Amino Acid Substitutions (AAS) Among SARS-CoV-2 Variants

Table 3. Comparison of Single Nucleotide Polymorphisms (SNP) and Resultant Amino Acid Substitutions (AAS) or Deletions in the Spike Gene Among SARS-CoV-2 Variants

ACCESSION AND IDENTIFIER	SNP and AAS																																	
	S13I		L18F		T20N		P26S		Q52R		A67V		ΔH69		ΔV70		D80A		T95I		D138Y		G142D		Y144		ΔY145		W152C		E154K		R190S	
	21,600 NT	13 AA	21,614 NT	18 AA	21,621 NT	20 AA	21,638 NT	26 AA	21,717 NT	52 AA	21,762 NT	67 AA	21,766-8 NT	69 AA	21,769-71 NT	70 AA	21,801 NT	80 AA	21,846 NT	95 AA	21,974 NT	138 AA	21,987 NT	142 AA	21,994 NT	144 AA	21,995-7 NT	145 AA	22,018 NT	152 AA	22,022 NT	154 AA	22,132 NT	190 AA
NC_045512_Reference Genome_Wuhan	G	Ser (S)	C	Leu (L)	C	Thr (T)	C	Pro (P)	A	Gln (Q)	C	Ala (A)	ACA	His (H)	TGT	Val (V)	A	Asp (D)	C	Thr (T)	G	Asp (D)	G	Gly (G)	T	Tyr (Y)	TAC	Tyr (Y)	G	Trp (W)	G	Glu (E)	G	Arg (R)
EPI_ISL_601443_UK_B.1.1.7_Alpha	G	Ser (S)	C	Leu (L)	C	Thr (T)	C	Pro (P)	A	Gln (Q)	C	Ala (A)	---	Δ	---	Δ	A	Asp (D)	C	Thr (T)	G	Asp (D)	G	Gly (G)	C	Tyr (Y)	---	Δ	G	Trp (W)	G	Glu (E)	G	Arg (R)
EPI_ISL_712081_South Africa_B.1.351_Beta	G	Ser (S)	C	Leu (L)	C	Thr (T)	C	Pro (P)	A	Gln (Q)	C	Ala (A)	ACA	His (H)	TGT	Val (V)	C	Ala (A)	C	Thr (T)	G	Asp (D)	G	Gly (G)	T	Tyr (Y)	TAC	Tyr (Y)	G	Trp (W)	G	Glu (E)	G	Arg (R)
EPI_ISL_729975_Nigeria_B.1.1	G	Ser (S)	C	Leu (L)	C	Thr (T)	C	Pro (P)	A	Gln (Q)	C	Ala (A)	ACA	His (H)	TGT	Val (V)	A	Asp (D)	C	Thr (T)	G	Asp (D)	G	Gly (G)	T	Tyr (Y)	TAC	Tyr (Y)	G	Trp (W)	G	Glu (E)	G	Arg (R)
EPI_ISL_616802_Denmark_B.1.1.298	G	Ser (S)	C	Leu (L)	C	Thr (T)	C	Pro (P)	A	Gln (Q)	C	Ala (A)	---	Δ	---	Δ	A	Asp (D)	C	Thr (T)	G	Asp (D)	G	Gly (G)	T	Tyr (Y)	TAC	Tyr (Y)	G	Trp (W)	G	Glu (E)	G	Arg (R)
EPI_ISL_942929_L452R_B.1.427/429_Epsilon	T	Ile (I)	C	Leu (L)	C	Thr (T)	C	Pro (P)	A	Gln (Q)	C	Ala (A)	ACA	His (H)	TGT	Val (V)	A	Asp (D)	C	Thr (T)	G	Asp (D)	G	Gly (G)	T	Tyr (Y)	TAC	Tyr (Y)	T	Cys (C)	G	Glu (E)	G	Arg (R)
EPI_ISL_792680_Brazil_B.1.1.248/P.1_Gamma	G	Ser (S)	T	Phe (F)	A	Asn (N)	T	Ser (S)	A	Gln (Q)	C	Ala (A)	ACA	His (H)	TGT	Val (V)	A	Asp (D)	C	Thr (T)	T	Tyr (Y)	G	Gly (G)	T	Tyr (Y)	TAC	Tyr (Y)	G	Trp (W)	G	Glu (E)	T	Ser (S)
EPI_ISL_918536_Brazil_P.2_Zeta	G	Ser (S)	C	Leu (L)	C	Thr (T)	C	Pro (P)	A	Gln (Q)	C	Ala (A)	ACA	His (H)	TGT	Val (V)	A	Asp (D)	C	Thr (T)	G	Asp (D)	G	Gly (G)	T	Tyr (Y)	TAC	Tyr (Y)	G	Trp (W)	G	Glu (E)	G	Arg (R)
EPI_ISL_1739895_B.1.525_Eta	G	Ser (S)	C	Leu (L)	C	Thr (T)	C	Pro (P)	G	Arg (R)	T	Val (V)	---	Δ	---	Δ	A	Asp (D)	C	Thr (T)	G	Asp (D)	G	Gly (G)	C	Tyr (Y)	---	Δ	G	Trp (W)	G	Glu (E)	G	Arg (R)
EPI_ISL_1372093_India_B.1.617.1_Kappa	G	Ser (S)	C	Leu (L)	C	Thr (T)	C	Pro (P)	A	Gln (Q)	C	Ala (A)	ACA	His (H)	TGT	Val (V)	A	Asp (D)	T	Ile (I)	G	Asp (D)	A	Asp (D)	T	Tyr (Y)	TAC	Tyr (Y)	G	Trp (W)	A	Lys (K)	G	Arg (R)
EPI_ISL_1663516_India_B.1.617.2_Delta	G	Ser (S)	C	Leu (L)	C	Thr (T)	C	Pro (P)	A	Gln (Q)	C	Ala (A)	ACA	His (H)	TGT	Val (V)	A	Asp (D)	C	Thr (T)	G	Asp (D)	G	Gly (G)	T	Tyr (Y)	TAC	Tyr (Y)	G	Trp (W)	G	Glu (E)	G	Arg (R)
SARS-CoV-2, Isolate USA-HI498/2020_B.1.243	G	Ser (S)	C	Leu (L)	C	Thr (T)	C	Pro (P)	A	Gln (Q)	C	Ala (A)	ACA	His (H)	TGT	Val (V)	A	Asp (D)	C	Thr (T)	G	Asp (D)	G	Gly (G)	T	Tyr (Y)	TAC	Tyr (Y)	G	Trp (W)	G	Glu (E)	G	Arg (R)

ACCESSION AND IDENTIFIER	SNP and AAS																																	
	L241		ΔL242		ΔA243		ΔL244		R246I		K417T		K417N		L452R		Y453F		T478K		E484K		E484Q		N501Y		A570D		D614G		H655Y		Q677H	
	22,284-5 NT	241 AA	22,286-8 NT	242 AA	22,289-91 NT	243 AA	22,292 NT	244 AA	22,299 NT	246 AA	22,812 NT	417 AA	22,813 NT	417 AA	22,917 NT	452 AA	22,920 NT	453 AA	22,995 NT	478 AA	23,012 NT	484 AA	23,012 NT	484 AA	23,063 NT	501 AA	23,271 NT	570 AA	23,403 NT	614 AA	23,525 NT	655 AA	23,593 NT	677 AA
NC_045512_Reference Genome_Wuhan	TA	Leu (L)	CTT	Leu (L)	GCT	Ala (A)	T	Leu (L)	G	Arg (R)	A	Lys (K)	G	Lys (K)	T	Leu (L)	A	Tyr (Y)	C	Thr (T)	G	Glu (E)	G	Glu (E)	A	Asn (N)	C	Ala (A)	A	Asp (D)	C	His (H)	G	Gln (Q)
EPI_ISL_601443_UK_B.1.1.7_Alpha	TA	Leu (L)	CTT	Leu (L)	GCT	Ala (A)	T	Leu (L)	G	Arg (R)	A	Lys (K)	G	Lys (K)	T	Leu (L)	A	Tyr (Y)	C	Thr (T)	G	Glu (E)	G	Glu (E)	T	Tyr (Y)	A	Asp (D)	G	Gly (G)	C	His (H)	G	Gln (Q)
EPI_ISL_712081_South Africa_B.1.351_Beta	--	Leu (L)*	---	Δ	---	Δ	-	Leu (L)*	T	Ile (I)	A	Lys (K)	T	Asn (N)	T	Leu (L)	A	Tyr (Y)	C	Thr (T)	A	Lys (K)	A	Lys (K)	T	Tyr (Y)	C	Ala (A)	G	Gly (G)	C	His (H)	G	Gln (Q)
EPI_ISL_729975_Nigeria_B.1.1	TA	Leu (L)	CTT	Leu (L)	GCT	Ala (A)	T	Leu (L)	G	Arg (R)	A	Lys (K)	G	Lys (K)	T	Leu (L)	A	Tyr (Y)	C	Thr (T)	G	Glu (E)	G	Glu (E)	A	Asn (N)	C	Ala (A)	G	Gly (G)	C	His (H)	G	Gln (Q)
EPI_ISL_616802_Denmark_B.1.1.298	TA	Leu (L)	CTT	Leu (L)	GCT	Ala (A)	T	Leu (L)	G	Arg (R)	A	Lys (K)	G	Lys (K)	T	Leu (L)	T	Phe (F)	C	Thr (T)	G	Glu (E)	G	Glu (E)	A	Asn (N)	C	Ala (A)	G	Gly (G)	C	His (H)	G	Gln (Q)
EPI_ISL_942929_L452R_B.1.427/429_Epsilon	TA	Leu (L)	CTT	Leu (L)	GCT	Ala (A)	T	Leu (L)	G	Arg (R)	A	Lys (K)	G	Lys (K)	G	Arg (R)	A	Tyr (Y)	C	Thr (T)	G	Glu (E)	G	Glu (E)	A	Asn (N)	C	Ala (A)	G	Gly (G)	C	His (H)	G	Gln (Q)
EPI_ISL_792680_Brazil_B.1.1.248/P.1_Gamma	TA	Leu (L)	CTT	Leu (L)	GCT	Ala (A)	T	Leu (L)	G	Arg (R)	C	Thr (T)	G	Lys (K)	T	Leu (L)	A	Tyr (Y)	C	Thr (T)	A	Lys (K)	A	Lys (K)	T	Tyr (Y)	C	Ala (A)	G	Gly (G)	T	Tyr (Y)	G	Gln (Q)
EPI_ISL_918536_Brazil_P.2_Zeta	TA	Leu (L)	CTT	Leu (L)	GCT	Ala (A)	T	Leu (L)	G	Arg (R)	A	Lys (K)	G	Lys (K)	T	Leu (L)	A	Tyr (Y)	C	Thr (T)	A	Lys (K)	A	Lys (K)	A	Asn (N)	C	Ala (A)	G	Gly (G)	C	His (H)	G	Gln (Q)
EPI_ISL_1739895_B.1.525_Eta	TA	Leu (L)	CTT	Leu (L)	GCT	Ala (A)	T	Leu (L)	G	Arg (R)	A	Lys (K)	G	Lys (K)	T	Leu (L)	A	Tyr (Y)	C	Thr (T)	A	Lys (K)	A	Lys (K)	A	Asn (N)	C	Ala (A)	G	Gly (G)	C	His (H)	C	His (H)
EPI_ISL_1372093_India_B.1.617.1_Kappa	TA	Leu (L)	CTT	Leu (L)	GCT	Ala (A)	T	Leu (L)	G	Arg (R)	A	Lys (K)	G	Lys (K)	G	Arg (R)	A	Tyr (Y)	C	Thr (T)	C	Gln (Q)	C	Gln (Q)	A	Asn (N)	C	Ala (A)	G	Gly (G)	C	His (H)	G	Gln (Q)
EPI_ISL_1663516_India_B.1.617.2_Delta	TA	Leu (L)	CTT	Leu (L)	GCT	Ala (A)	T	Leu (L)	G	Arg (R)	A	Lys (K)	G	Lys (K)	G	Arg (R)	A	Tyr (Y)	A	Lys (K)	C	Gln (Q)	G	Glu (E)	A	Asn (N)	C	Ala (A)	G	Gly (G)	C	His (H)	G	Gln (Q)
SARS-CoV-2, Isolate USA-HI498/2020_B.1.243	TA	Leu (L)	CTT	Leu (L)	GCT	Ala (A)	T	Leu (L)	G	Arg (R)	A	Lys (K)	G	Lys (K)	T	Leu (L)	A	Tyr (Y)	C	Thr (T)	G	Glu (E)	G	Glu (E)	A	Asn (N)	C	Ala (A)	G	Gly (G)	C	His (H)	G	Gln (Q)

ACCESSION AND IDENTIFIER	SNP and AAS																																	
	P681H		P681R		I692V		A701V		T716I		G838		A845S		F888L		S929		D950N		S982A		T1027I		Q1071H		D1118H		D1146		V1176F		M1229I	
	23,604 NT	681 AA	23,604 NT	681 AA	23,636 NT	692 AA	23,644 NT	701 AA	23,709 NT	716 AA	24,076 NT	838 AA	24,095 NT	845 AA	24,224 NT	888 AA	24,349 NT	929 AA	24,410 NT	950 AA	24,506 NT	982 AA	24,642 NT	1027 AA	24,775 NT	1071 AA	24,914 NT	1118 AA	25,000 NT	1146 AA	25,088 NT	1176 AA	25,249 NT	1229 AA
NC_045512_Reference Genome_Wuhan	C	Pro (P)	C	Pro (P)	A	Ile (I)	C	Ala (A)	C	Thr (T)	T	Gly (G)	G	Ala (A)	T	Phe (F)	T	Ser (S)	G	Asp (D)	T	Ser (S)	C	Thr (T)	A	Gln (Q)	G	Asp (D)	C	Asp (D)	G	Val (V)	G	Met (M)
EPI_ISL_601443_UK_B.1.1.7_Alpha	A	His (H)	A	His (H)	A	Ile (I)	C	Ala (A)	T	Ile (I)	T	Gly (G)	G	Ala (A)	T	Phe (F)	T	Ser (S)	G	Asp (D)	G	Ala (A)	C	Thr (T)	A	Gln (Q)	C	His (H)	C	Asp (D)	G	Val (V)	G	Met (M)
EPI_ISL_712081_South Africa_B.1.351_Beta	C	Pro (P)	C	Pro (P)	A	Ile (I)	T	Val (V)	C	Thr (T)	T	Gly (G)	G	Ala (A)	T	Phe (F)	T	Ser (S)	G	Asp (D)	T	Ser (S)	C	Thr (T)	A	Gln (Q)	G	Asp (D)	C	Asp (D)	G	Val (V)	G	Met (M)
EPI_ISL_729975_Nigeria_B.1.1	A	His (H)	A	His (H)	A	Ile (I)	C	Ala (A)	C	Thr (T)	T	Gly (G)	T	Ser (S)	T	Phe (F)	T	Ser (S)	G	Asp (D)	T	Ser (S)	C	Thr (T)	A	Gln (Q)	G	Asp (D)	C	Asp (D)	G	Val (V)	G	Met (M)
EPI_ISL_616802_Denmark_B.1.1.298	C	Pro (P)	C	Pro (P)	G	Val (V)	C	Ala (A)	C	Thr (T)	T	Gly (G)	G	Ala (A)	T	Phe (F)	T	Ser (S)	G	Asp (D)	T	Ser (S)	C	Thr (T)	A	Gln (Q)	G	Asp (D)	T	Asp (D)	G	Val (V)	T	Ile (I)
EPI_ISL_942929_L452R_B.1.427/429_Epsilon	C	Pro (P)	C	Pro (P)	A	Ile (I)	C	Ala (A)	C	Thr (T)	T	Gly (G)	G	Ala (A)	T	Phe (F)	C	Ser (S)	G	Asp (D)	T	Ser (S)	C	Thr (T)	A	Gln (Q)	G	Asp (D)	C	Asp (D)	G	Val (V)	G	Met (M)
EPI_ISL_792680_Brazil_B.1.1.248/P.1_Gamma	C	Pro (P)	C	Pro (P)	A	Ile (I)	C	Ala (A)	C	Thr (T)	T	Gly (G)	G	Ala (A)	T	Phe (F)	T	Ser (S)	G	Asp (D)	T	Ser (S)	T	Ile (I)	A	Gln (Q)	G	Asp (D)	C	Asp (D)	T	Phe (F)	G	Met (M)
EPI_ISL_918536_Brazil_P.2_Zeta	C	Pro (P)	C	Pro (P)	A	Ile (I)	C	Ala (A)	C	Thr (T)	T	Gly (G)	G	Ala (A)	T	Phe (F)	T	Ser (S)	G	Asp (D)	T	Ser (S)	C	Thr (T)	A	Gln (Q)	G	Asp (D)	C	Asp (D)	T	Phe (F)	G	Met (M)
EPI_ISL_1739895_B.1.525_Eta	C	Pro (P)	C	Pro (P)	A	Ile (I)	C	Ala (A)	C	Thr (T)	T	Gly (G)	G	Ala (A)	C	Leu (L)	T	Ser (S)	G	Asp (D)	T	Ser (S)	C	Thr (T)	A	Gln (Q)	G	Asp (D)	C	Asp (D)	G	Val (V)	G	Met (M)
EPI_ISL_1372093_India_B.1.617.1_Kappa	G	Arg (R)	G	Arg (R)	A	Ile (I)	C	Ala (A)	C	Thr (T)	T	Gly (G)	G	Ala (A)	T	Phe (F)	T	Ser (S)	G	Asp (D)	T	Ser (S)	C	Thr (T)	T	His (H)	G	Asp (D)	C	Asp (D)	G	Val (V)	G	Met (M)
EPI_ISL_1663516_India_B.1.617.2_Delta	G	Arg (R)	G	Arg (R)	A	Ile (I)	C	Ala (A)	C	Thr (T)	T	Gly (G)	G	Ala (A)	T	Phe (F)	T	Ser (S)	A	Asn (N)	T	Ser (S)	C	Thr (T)	A	Gln (Q)	G	Asp (D)	C	Asp (D)	G	Val (V)	G	Met (M)
SARS-CoV-2, Isolate USA-HI498/2020_B.1.243	A	His (H)	A	His (H)	A	Ile (I)	C	Ala (A)	C	Thr (T)	C	Gly (G)	G	Ala (A)	T	Phe (F)	T	Ser (S)	G	Asp (D)	T	Ser (S)	C	Thr (T)	A	Gln (Q)	G	Asp (D)	C	Asp (D)	G	Val (V)	G	Met (M)

*combine to code for one Leucine

Data Availability Statement: Tables of acknowledgements for the genome sequences and data from GISAID, as well as data sets used in these analyses, are available at: https://github.com/dpmaison/Algorithm-for-the-Quantitation-of-Variants-of-Concern-for-Rationally-Designed-Vaccines. The viral genome sequences used in this publication are publicly available from GenBank (https://www.ncbi.nlm.nih.gov/sars-cov-2/) and GISAID (https://gisaid.org). Other genome sequences from GISAID are referenced in-text. GenBank Accession numbers for sequencing data are: SARS-CoV-2, isolate USA-HI498 2020 (Stock Virus: MZ664037) (VTM: OK021552) and SARS-CoV-2, isolate USA-HI708 2020 (Stock Virus: MZ664038) (VTM: OK189251).
