# Supplementary material for: Dynamic SARS-CoV-2 emergence algorithm for rationally-designed logical next-generation vaccines
Source: Commun Biol. 2022 Oct 10;5:1081. doi: 10.1038/s42003-022-04030-3 (PMC9550860; doi:10.1038/s42003-022-04030-3)
Supplement: Supplementary file 3 — Description of Additional Supplementary Files [file 42003_2022_4030_MOESM3_ESM.pdf]

Description of Additional Supplementary Files

File name: Supplementary Data 1
Description: source data used to generate Figure 1e-m, Figure 2, Figure 3, Figure 5, Supplementary Figure 1, and Supplementary Table 2
