# Supplementary material for: Dynamic SARS-CoV-2 emergence algorithm for rationally-designed logical next-generation vaccines
Source: Commun Biol. 2022 Oct 10;5:1081. doi: 10.1038/s42003-022-04030-3 (PMC9550860; doi:10.1038/s42003-022-04030-3)
Supplement: Supplementary file 5 — Reporting Summary [file 42003_2022_4030_MOESM5_ESM.pdf]

Corresponding author(s): Vivek R. Nerurkar

Last updated by author(s): Sep 8, 2022

Reporting Summary

Nature Portfolio wishes to improve the reproducibility of the work that we publish. This form provides structure for consistency and transparency in reporting. For further information on Nature Portfolio policies, see our Editorial Policies and the Editorial Policy Checklist.

Statistics

For all statistical analyses, confirm that the following items are present in the figure legend, table legend, main text, or Methods section.

n/a	Confirmed	
☐	☒	The exact sample size (n) for each experimental group/condition, given as a discrete number and unit of measurement
☒	☐	A statement on whether measurements were taken from distinct samples or whether the same sample was measured repeatedly
☐	☒	The statistical test(s) used AND whether they are one- or two-sided<br>*Only common tests should be described solely by name; describe more complex techniques in the Methods section.*
☐	☒	A description of all covariates tested
☒	☐	A description of any assumptions or corrections, such as tests of normality and adjustment for multiple comparisons
☐	☒	A full description of the statistical parameters including central tendency (e.g. means) or other basic estimates (e.g. regression coefficient) AND variation (e.g. standard deviation) or associated estimates of uncertainty (e.g. confidence intervals)
☐	☒	For null hypothesis testing, the test statistic (e.g. F, t, r) with confidence intervals, effect sizes, degrees of freedom and P value noted<br>*Give P values as exact values whenever suitable.*
☒	☐	For Bayesian analysis, information on the choice of priors and Markov chain Monte Carlo settings
☒	☐	For hierarchical and complex designs, identification of the appropriate level for tests and full reporting of outcomes
☒	☐	Estimates of effect sizes (e.g. Cohen's d, Pearson's r), indicating how they were calculated

Our web collection on statistics for biologists contains articles on many of the points above.

Software and code

Policy information about availability of computer code

Data collection	Data collection was utilized using the specified workflow, commercial processes, and publicly available databases.
Data analysis	Data analysis was conducted using RStudio 1.3.1093 (with ggplot2 package), GraphPad Prism 9 Version 9.2.0, and SnapGene Version 5.3.2. Lineage assignment was done using Pangolin Web Application.

For manuscripts utilizing custom algorithms or software that are central to the research but not yet described in published literature, software must be made available to editors and reviewers. We strongly encourage code deposition in a community repository (e.g. GitHub). See the Nature Portfolio guidelines for submitting code & software for further information.

Data

Policy information about availability of data

All manuscripts must include a data availability statement. This statement should provide the following information, where applicable:

- Accession codes, unique identifiers, or web links for publicly available datasets
- A description of any restrictions on data availability
- For clinical datasets or third party data, please ensure that the statement adheres to our policy

The viral genome sequences used in this publication are publicly available from GenBank (https://www.ncbi.nlm.nih.gov/sars-cov-2/) and GISAID (https://gisaid.org). Tables of acknowledgments for the genome sequences from GISAID are available at: https://github.com/dpmaison/Algorithm-for-the-Quantitation-of-Variants-of-Concern-for-Rationally -Designed-Vaccines. Other genome sequences from GISAID are referenced in-text.

Field-specific reporting

Please select the one below that is the best fit for your research. If you are not sure, read the appropriate sections before making your selection.

☒ Life sciences ☐ Behavioural & social sciences ☐ Ecological, evolutionary & environmental sciences

For a reference copy of the document with all sections, see nature.com/documents/nr-reporting-summary-flat.pdf

Life sciences study design

All studies must disclose on these points even when the disclosure is negative.

Sample size	Sample sizes are based on the number of sequences reported in GISAID corresponding to a specific SARS-CoV-2 variant of concern. Hawaii sequences are all available sequences from GISAID and GenBank.
Data exclusions	Sequences with ambiguous nucleotides were excluded from whole-genome phylogenetic analysis per previously established workflow.
Replication	Data were collected, analyzed, recollected, and reanalyzed to ensure replication.
Randomization	Randomization is not relevant to our study because groups are assigned via established Pangolin phylogeny for SARS-CoV-2 (utilized by both CDC and WHO).
Blinding	Blinding is not relevant to our study because awareness of group assignment does not affect collection or analysis.

Reporting for specific materials, systems and methods

We require information from authors about some types of materials, experimental systems and methods used in many studies. Here, indicate whether each material, system or method listed is relevant to your study. If you are not sure if a list item applies to your research, read the appropriate section before selecting a response.

Materials & experimental systems

n/a	Involved in the study	
☒	☐	Antibodies
☐	☒	Eukaryotic cell lines
☒	☐	Palaeontology and archaeology
☒	☐	Animals and other organisms
☐	☒	Human research participants
☐	☒	Clinical data
☒	☐	Dual use research of concern

Methods

n/a	Involved in the study	
☒	☐	ChIP-seq
☒	☐	Flow cytometry
☒	☐	MRI-based neuroimaging

Eukaryotic cell lines

Policy information about cell lines

Cell line source(s)	ATCC (CRL-1586)
Authentication	Cell lines authenticated by ATCC.
Mycoplasma contamination	Cell lines were verified by ATCC.
Commonly misidentified lines (See ICLAC register)	N/A

Human research participants

Policy information about studies involving human research participants

Population characteristics	Both 498 and 708 patients were males, one is Caucasian and the other Japanese/Okinawan/Filipino, and their mean age is 29.5 years.
Recruitment	patients qRT-PCR positive for SARS-CoV-2 were recruited.
Ethics oversight	University of Hawaii at Manoa IRB (#2020-00367)

Note that full information on the approval of the study protocol must also be provided in the manuscript.

Clinical data

Policy information about clinical studies

All manuscripts should comply with the ICMJE guidelines for publication of clinical research and a completed CONSORT checklist must be included with all submissions.

Clinical trial registration	#NCT04360551
Study protocol	https://clinicaltrials.gov/ct2/show/NCT04360551?term=04360551&draw=2&rank=1
Data collection	Settings were the University of Hawaii - Hawaii Center for AIDS and patient homes. The time was August 2020.
Outcomes	Pre-defined primary and secondary outcomes are qRT-PCR positivity.
